# Supplementary material for: Thermochemiluminescent peroxide crystals
Source: Nat Commun. 2019 Mar 1;10:997. doi: 10.1038/s41467-019-08816-8 (PMC6397279; doi:10.1038/s41467-019-08816-8)
Supplement: Supplementary file 1 — Supplementary Information [file 41467_2019_8816_MOESM1_ESM.pdf]

SUPPLEMENTARY INFORMATION

Thermochemiluminescent Peroxide Crystals

Schramm et al.

Contents

Supplementary Methods **3**

1. Experimental details 3
   - 1.1. Synthesis 3
   - 1.2. Design and technical details of the RGB photoreactor 3
   - 1.3. Isolation of the decomposition products 4
   - 1.4. X-ray diffraction analysis 4
   - 1.5. Variable-temperature μIR spectroscopy 5
   - 1.6. Mass spectrometry 5
   - 1.7. Optical spectroscopy 5
   - 1.8. Chemiluminescence quantum yield measurements 6
   - 1.9. Computed tomography (CT) 7
   - 1.10. Powder X-ray diffraction 7
   - 1.11. Thermal analysis 7
   - 1.12. Scanning electron microscopy 8
   - 1.13. Low-light microscopy 8
2. Computational Details 8
   - 2.1. General details 8
   - 2.2. IBO calculations 8
   - 2.3. ESP calculations 8

Supplementary Tables **9**

Supplementary Figures **15**

Supplementary References **24**

Supplementary Methods

1. Experimental details

1.1. Synthesis

The chemicals were purchased from Sigma Aldrich and the solvents for synthesis were purchased from Fisher and used as obtained.

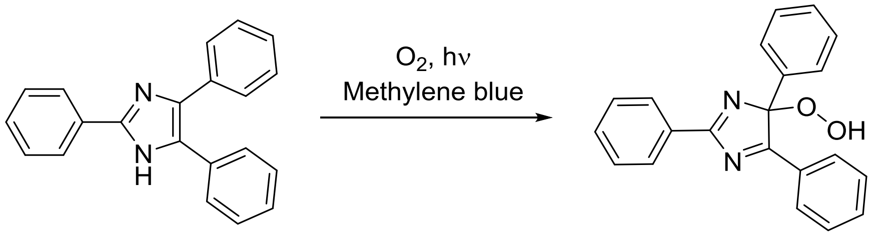

Lophine hydroperoxide was synthesized by Schenck-ene reaction from lophine (2,4,5-triphenyl-1*H*-imidazole) and singlet oxygen. The singlet oxygen was generated *in situ*, by reaction of dissolved triplet oxygen and photosensitized methylene blue. 2 g lophine were dissolved in about 50 mL of 9:1 mixture of dichloromethane and carbon disulfide and cooled to –10 °C. In the meantime, about 10 mg of methylene blue were dissolved in approximately 10 mL dichloromethane. Silica gel was added to the solution until it was completely discolored (ca. 10 g) to adsorb the methylene blue. The silica gel was filtered and about 1 g of it was suspended in the lophine solution with strong stirring using magnetic stirrer. Under a continuous stream of oxygen (ca. 1 bubble/second) the reaction solution was irradiated with red (630 nm) LED light. The progress of the reaction was monitored with TLC and the irradiation was stopped after the reaction was completed. The silica particles were filtered of the still cold (~ –10 °C) solution. The solvent was slowly evaporated. TGA studies (see Section 1.11) indicated that LHP is stable at room temperature with years, and significant decomposition can only be detected over 50 °C. Thus, it is recommend that all workup procedures are performed below 50 °C. Recrystallization of the crude product was attempted from various solvents (CS_2, ethyl acetate, THF, DMF, dioxane, DCM). Best results were obtained by crystallization in a freezer (–33 °C) from ethanol as colorless large (0.1–1 cm) block-shaped crystals. Yield: 78% (after recrystallization). The pure reaction product thermally decomposes at ca. 110–115 °C. ^{1}H NMR (500 MHz, chloroform-*d*) δ 13.88 (s, 2H), 8.44 – 8.34 (m, 2H), 8.00 – 7.90 (m, 2H), 7.63 – 7.56 (m, 1H), 7.56 – 7.44 (m, 4H), 7.37 – 7.29 (m, 4H), 7.22 (t, J = 7.8 Hz, 2H). ^{13}C NMR (126 MHz, chloroform-*d*), δ (ppm): 191.16, 172.32, 134.78, 134.44, 133.09, 132.14, 130.58, 130.12, 129.76, 129.34, 128.92, 128.67, 128.29, 126.54, 115.28. HR-ESI-TOF-MS *m/z* (%): calc. 329.1285 [$C_{21}H_{17}N_2O_2^+$], exp. 329.1296 (100) [M+H].

1.2. Design and technical details of the RGB photoreactor

The photooxygenation procedure described in the previous section was carried out by using a custom-built RGB photoreactor. Two flexible Adafruit DotStar 8×32 256 RGB LED matrices were bend in a U-shape around a reaction flask and embedded in a 3D-printed frame. The LEDs were controlled via an Arduino Uno and cooled with three 120 mm-fans. The color of the light emitted by the photoreactor can be controlled to cover the entire visible spectral range. Since the synthesis of lophine hydroperoxide required methylene blue as sensitizer dye, red light with wavelength of 630 nm was used for excitation.

1.3. Isolation of the decomposition products

1 g of lophine hydroperoxide was dissolved in 50 mL boiling toluene that resulted in immediate evolution of oxygen. The solution was stirred under reflux for another 30 min. After about 45 min stirring the reaction product started to precipitate as long colorless needles. The reaction mixture was allowed to cool to room temperature, and the reaction product was filtered. After drying, the precipitate was identified as lophine by ^{1}H NMR and MS spectroscopy (^{1}H NMR (500 MHz, DMSO-d_6) δ (ppm): 12.73 (s, 1H), 8.08 (d, J = 7.7 Hz, 2H), 7.56–7.26 (m, 13H), 7.22 (t, J = 7.3 Hz, 1H). HR-ESI-TOF-MS m/z (%): calc. 297.1386 [$C_{21}H_{17}N_2^+$], exp. 297.1424 (100) [M+H])). The product identity was confirmed by crystal structure determination. The precipitate had a mass of ~500 mg and was of analytical purity (UHPLC). Analytical UHPLC analysis was also carried out on the remaining product after thermal decomposition of a solid sample of LHP using an Agilent Technologies 1290 Infinity II system equipped with an Agilent EclipisePlusC18 2.1 × 50 mm column with 1.8 µm silica beads together with an Agilent EclipisePlusC18 2.1 × 5 mm guard column with 1.8 µm silica beads. The UHPLC runs were carried out by using a linear gradient of 95% water to 100% acetonitrile over a time period of 10 min with a flow rate of 0.3 mL min^{-1}, a maximum pressure of 600 bar and diode array detector. The decomposition sample contained a significant amount of lophine as well as minor traces of side-products. Side-product 1 was identified by high resolution mass spectrometry as dibenzoylamidine, which is in line with earlier results[1] for the reaction in solution. Attempts to isolate this side-product by preparative liquid chromatography resulted in its hydrolysis to *N*-benzoylbenzamide, which was identified by ^{1}H NMR and mass spectrometry (^{1}H NMR (500 MHz, chloroform-d) δ (ppm): 8.88 (s, 1H), 7.90–7.84 (m, 4H), 7.62 (t, J = 7.5 Hz, 2H), 7.52 (t, J = 7.6 Hz, 4H). HR-ESI-TOF-MS m/z (%): calc. 226.0863 [$C_{14}H_{12}NO_2^+$], exp. 226.0869 (100) [M+H]) and single crystal X-ray diffraction.

1.4. X-ray diffraction analysis

The X-ray diffraction data were collected by using a Bruker APEX DUO diffractometer equipped with a Cobra cooling device (Oxford Cryosystems) with graphite-monochromated MoK_α radiation (λ = 0.71073 Å) and CuK_α radiation (λ = 1.54178 Å) and CCD as area detector. Data collection, integration, scaling and absorption corrections were performed by using Bruker Apex II software[2]. The integration and scaling of the data were performed by using the program SAINT[3]. The X-ray diffraction data were corrected for absorption effects using SADABS[4]. The structure was solved by direct methods, implemented in SHELXS-97[5]. The structure refinement, using the OLEX2 interface[6] was performed by using the full-matrix least-squares method, based on F^2 against all reflections as implemented in SHELXL-2014/7. The hydrogen atoms bonded to carbon atoms were fixed using the HFIX command in SHELX-TL. The graphics for publication were generated using Mercury 3.7[7], X-Seed[8], and POV-Ray[9].

1.5. Variable-temperature μIR spectroscopy

Infrared spectra were recorded at BL43IR beamline SPring-8 (Japan). A Vertex70 FTIR spectrometer equipped with a Hyperion 2000 infrared microscope (Bruker) and MCT detector was used. All spectra were recorded in the mid-IR range (800–4000 cm^{-1}) with 2 cm^{-1} resolution and 256-fold accumulation. The IR synchrotron radiation was not spatially constrained and it was focused normal to the crystal plane of ~30-μm-thick LHP crystal. The variable-temperature measurements were performed by using a Linkam hot stage with liquid-nitrogen cooling system.

1.6. Mass spectrometry

The spectra were recorded on electrospray quadrupole/time-of-flight-type mass spectrometer MicrOTOF-Q (Bruker Daltonics, Germany) operated in positive ion mode. The performance and resolution were verified using Tunemix (Agilent Technologies, USA) with resolution of *m*/*z* 122 of 12000. Mass calibration was achieved using Tunemix (internal calibration) between *m*/*z* 50 and 3000 in the same acquisition mode that affords accuracy of 10 ppm.

1.7. Optical spectroscopy

All chemiluminescence, fluorescence and phosphorescence spectra, as well as the kinetics were recorded on a Jasco FP8500 spectrofluorimeter equipped with either a ILFC-847S cooled integration sphere and ESC-842 reference light source for the quantum yield and for the low-temperature measurements, or with ETC-815 temperature cell for the kinetic measurements. Solid-state absorbance measurements were carried out with the same instrument utilizing a FUV-803 absorbance cell. Solution-state absorbance measurements were carried out on a Shimadzu UV-3600 UV-VIS-NIR spectrophotometer. The solvents used for UV-Vis and emission spectroscopy were of analytical grade and purchased from Fisher. The photoluminescence spectra of lophine were recorded at –196 °C in order to extend the lifetime of the excited triplet state and to be able to record a time-gated phosphorescence spectrum. We note that in order to observe light emission, the LHP crystals have to be heated to their decomposition temperature (116.5 °C). Heating below this temperature does not result in any detectable emission of light (Supplementary Figure 14). The kinetic trace of the thermochemiluminesce reaction follows first order kinetics ($R^2 > 0.99$). The rate constants were derived from this model (Supplementary Figure 15, Supplementary Table 4). The activation parameters (Supplementary Table 5) were also derived from the isothermal chemiluminescence kinetics which were analyzed with the Arrhenius equation (Supplementary Figure 16):

$$\ln(k) = \ln(A) - \frac{E_a}{R}\left(\frac{1}{T}\right) \qquad (1)$$

and with the Eyring equation:

$$\frac{\ln k}{T} = \frac{-\Delta H^{\ddagger}}{R} \cdot \frac{1}{T} + \ln\frac{k_B}{h} + \frac{\Delta S^{\ddagger}}{R} \qquad (2)$$

The results of deconvolution of the two-band spectra presented in Figure 3 in the main text are given in Supplementary Table 3. Typical example of spectral deconvolution is presented in Supplementary Figure 8 which shows the spectral maxima of high- and low-energy emission bands, as well as their relative intensities. The Kamlet-Taft solvent parameters are also listed in Supplementary Table 3.

In the case of polar chromophore capable of acid-base interactions, one of the best analytical methods to describe the solvent dependence of various solute parameters is the Kamlet-Taft (KT) approach[10] that takes into account the non-specific (dipole solvation) and specific (point-to-point acid-base interactions) solvation effects. In this method, the solvents are characterized by their dipole solvation ability (π*), as well as the ability to donate (α) and accept (β) protons from the solute.

We fitted the solvatochromic behavior and the intensity ratio of the emission bands by using the KT method. The high-energy emission band maximum varied in a very narrow range (424–432 nm), and its analysis resulted in a regression with a poor correlation coefficient. The KT analysis of the low-energy band was more satisfactory and resulted in the correlation:

$$\nu_B(1000/\mathrm{cm}) = 19.1 - 0.6\beta + 0.7\pi^* \ (R = 0.86) \qquad (3)$$

where $\nu_B = 10^7 / \lambda_B$ (nm). These results show that increase of solvent basicity and decrease of solvent polarity lead to bathochromic shift of this band. The I_A / I_B ratio also demonstrated a satisfactory correlation (the NMP data are excluded):

$$I_A / I_B = 1.0 - 1.2\beta + 0.7\pi^* \ (R = 0.85) \qquad (4)$$

The result shows that increase of solvent basicity and decrease of solvent polarity lead to domination of the low-energy B-band, the basicity being a dominating factor, as it is inferred from the 1.2 and 0.7 solute susceptibility parameters.

1.8. Chemiluminescence quantum yield measurements

A Jasco FP8500 spectrofluorimeter equipped with either a ILFC-847S cooled Integration sphere and ESC-842 reference light source was used for the chemiluminescence quantum yield measurements. The instrument was coupled to additional equipment, as is shown schematically in Supplementary Figure 9. A crystalline sample of LHP in a ceramic pan was placed in the micro-heating stage of an integrating sphere, as shown in Supplementary Figure 9. The micro-heating stage was controlled by a controller (Unisoku) and regulated by the internal thermistor which was placed in close proximity to the sample in the micro-heating stage. After the desired temperature was reached, the chemiluminescence signal was recorded by using Jasco FP-8500 fluorescence spectrometer (time resolution of up to 0.01 s). For the determination of the quantum yield of the sample, an LED was placed inside the integrating sphere instead of the crystalline sample. The LED had an emission maximum of ~540 nm in order to avoid wavelength-dependent response effects of the detector. The intensity of the LED was controlled with Thorlabs DC2200 LED Controller. The calibrated detector Thorlabs S130VC was used in combination with the Thorlabs PM320E Controller to record the irradiation power (in W cm^{-2}) of the LED. Knowing the inner surface of the Integrating sphere (314.16 cm^2), the total irradiation power of the LED was calculated. At the same time, the relative irradiation

of the LED (in RLU) was measured with the fluorescence spectrometer. The total irradiation power of the LED determined earlier was used to calibrate the *y*-axis from RLU into watts. After this procedure, the sample was measured. The resulting integral of the light emitted was converted from W s into photons by using $E = hc/\lambda$. The number of photons divided by the number of molecules (determined by the weight and the molar mass of the sample) equals the chemiluminescence quantum yield. The calibration of the setup was done with $n = 21$ to ensure statistical reliability. The determination of the actual quantum yield was done with $n = 10$. This resulted in a chemiluminescence quantum yield of $2.13 \times 10^{-7} \pm 1.07 \times 10^{-8}$ E mol^{-1}.

1.9. Computed tomography (CT)

The CT scans were acquired using an X View CT Scanner, X500 CT. Scans were taken with a working voltage of 120 kV and 100 A. The crystals were attached to a molding clay and scanned at a rate of 7.5 frames per second. The images were processed and videos were recorded using X View CT's default software, eFX-view. In the images with the CT scans, the density of the material is represented by its color, and locations with varying density have varying colors. The molding clay used to mount the sample was removed from the CT images for clarity, but can be seen in Supplementary Movie 5 as an attached blue mass.

1.10. Powder X-ray diffraction

Temperature-dependent powder X-ray diffraction (PXRD) data were collected with an Anton Paar high-temperature chamber HTK 1200N on Panalytical Empyrean system with Cu irradiation. Continuous scan with 0.013^{o} step size was applied. The sample was measured every 10 °C from 30 °C to 150 °C (every 2 °C from 70 °C to 110 °C). The starting material retained its structure up to about 100 °C. The reaction resulted in changes between 100 °C and 110 °C, and the product is stable till the end of experiment.

1.11. Thermal analysis

The thermal decomposition profile was investigated by simultaneous thermal analysis (TG and DTA) using Q600 STA analyzer (TA Instruments), from room temperature to 300 °C and with heating rate 10 °C min^{-1}. To assess the stability of LHP at room temperature, the isothermal rate of decomposition was also determined in 10 ^{o}C-intervals between 50 °C and 80 °C. No detectable decomposition could be detected below 50 °C even after 48 hours, the longest time attainable with our experimental setup. Instead, the decomposition rate at room temperature was determined by extrapolation of the decomposition rates measured at elevated temperatures by using the van't Hoff's rule:

$$Q_{10} = \left(\frac{k_2}{k_1}\right)^{\frac{10\ \mathrm{K}}{T_1 - T_2}} \quad (5)$$

with Q_{10} as the factor whose reaction (decomposition) time, increases with a 10 ^{o}C-increment. The values in Supplementary Table 6 were obtained. From this data, the average Q_{10} was calculated, $Q_{ave} = 4.28 \pm 2.29$. With $k_1 = k_{25\,°C}$, $k_2 = k_{50\,°C}$, $T_2 = 50$ °C and $T_1 = 25$ °C, the average decomposition rate at room temperature was estimated to $k_{25\,°C} = 4.86 \times 10^{-8} \pm 2.31 \times 10^{-8}$ min^{-1}. This rate constant corresponds to half-lifetime of $t_{1/2} = 27.2 \pm 12.9$ years. This lifetime indicates that LHP is sufficiently thermally stable to be considered for practical applications in the future.

1.12. Scanning electron microscopy

Temperature-controlled electron microscopy was performed using a Thermo Quanta 450 FEG scanning electron microscope. The normal sample stage was replaced with the Thermo 1000 °C stage. Imaging was performed in environmental mode (ESEM) with a 60 Pa atmosphere of deionized water using a gaseous secondary electron detector (GSED). The sample was loaded onto a graphite crucible with a working distance of ~20 mm and heated from room temperature to 160 °C while recording SEM micrographs. The LHP crystals decompose at a temperature of ~110–120 °C and expand in volume. Supplementary Movie 7 illustrates this process.

1.13. Low-light microscopy

In order to visualize the light emission of small crystals of LHP, a low-light microscope was constructed similar to that described by Pratx and co-workers[11,12]. The components that were used in the microscope are described in Supplementary Figure 12. The entire setup was encased in a custom-built absolutely light-sealed housing. Supplementary Movies 1–3 were recorded by using this setup. Supplementary Movie 4 was recorded by using a Canon EOS 6D DSLR camera with a Tamron 90 mm f 1:2.8 lens from the side of the heating stage.

2. Computational Details

2.1. General details

Density Functional Theory (DFT) calculations were performed with Gaussian G09 program package[S13] and visualized with GaussView5[14]. The ground-state geometries were obtained at the DFT B3LYP/6-31+G(d,p)[15,16] level of theory. The optimized ground states were verified by frequency calculation. The IBO calculations and analysis were performed by using the PBE functional, the def2-TVVP basis set and the univ-JFIT fit basis as implemented in IBOview v20150427[17,18].

2.2. IBO calculations

IBO calculations were carried out on an optimized (B3LYP / 6-31+G(d,p)) ground-state structure of neutral and deprotonated lophine molecule. The IBO charges were extracted for all atoms in the central imidazole core.

2.3. ESP calculations

In order to visualize the electrostatic potential (ESP) of lophine and the lophine anion, the total density and ESP cubes were calculated using GaussView5 on a medium grid based on the optimized (B3LYP / 6-31+G(d,p)) ground state structure. The ESP was mapped on the total density with an isovalue of for the electrostatic density of 0.0004. In the color-coded scheme, the colors are linearly distributed, red to blue, from -2.0×10^{-2} to 2.5×10^{-2} for lophine and -0.15 to -7.0×10^{-2} for the lophine anion.

Supplementary Tables

Supplementary Table 1. Crystallographic parameters and refinements of LHP, lophine and *N*-benzoylbenzamide

Parameters	Lophine	Lophine peroxide	*N*-benzoylbenzamide
Temperature / K	100	296	296
Radiation	Cu	Mo	Mo
Formula weight	296.4	328.4	225.2
Crystal system	Orthorhombic	Triclinic	Orthorhombic
Space group	$Pna2_1$	$P\bar{1}$	*Iba2*
a / Å	8.9021(21)	10.3976(17)	15.5990(12)
b / Å	11.8159(14)	10.8089(17)	8.3990(6)
c / Å	30.1100(36)	17.2704(27)	8.9086(7)
α / °	90	93.629(3)	90
β / °	90	90.769(3)	90
γ / °	90	117.137(3)	90
Volume / Å^3	31.67.16(7)	1721.88(31)	1167.17(15)
Z	8	4	8
Density / (g cm^{-3})	1.24	1.27	1.28
μ / mm^{-1}	0.567	0.083	0.087
F_{000}	1248.0	688.0	472.0
h_{min}, h_{max}	-10, 9	-12, 12	-22, 22
k_{min}, k_{max}	-14, 13	-13, 13	-12, 12
l_{min}, l_{max}	-35, 35	-21, 21	-13, 13
No. of measured reflections	22642	40606	14237
No. of unique reflections	5357	6775	1981
No. of reflections used	4981	4662	1886
R_{all}, R_{obs}	0.082, 0.079	0.080, 0.047	0.042, 0.038
$wR_{2,all}$, $wR_{2,obs}$	0.212, 0.210	0.120, 0.106	0.098, 0.095
$\Delta\rho_{min,max}$ / (e Å^{-3})	-0.391, 0.607	-0.159, 0.278	-0.191, 0.367
GooF	1.068	1.067	1.070
CCDC No.	1885196	1874149	1874147

Supplementary Table 2. Assignment of the experimental to the theoretical calculated vibrations and kinetic constants of lophine determined from the individual bands (the spectra and ordinal numbers of selected bands are shown in Supplementary Figure 7)

No.	$\tilde{\nu}_{Exp}$ / cm^{-1}	$\tilde{\nu}_{Theor}$ / cm^{-1}	Main vibrational modes	*k* / s^{-1}
8	915	932	γ(C—H$_{\text{out of plane}}$)	(1.54 ± 0.37) × 10^{-3}
7	965	989	δ(C—N—C)	(3.44 ± 0.19) × 10^{-3}
6	1233	1235	ν_{as}(N—C=C—N)	(2.23 ± 0.08) × 10^{-3}
5	1368	1261	ν_{s}(N—C=C—N)	(2.95 ± 0.33) × 10^{-3}
4	1410	1417	ν_{as}(amidine)	(3.31 ± 0.10) × 10^{-3}
3, 2	1518	1523, 1538	ν_{s}(C—N), ν_{as}(C=C$_{Arom}$)	(3.64 ± 0.18) × 10^{-3}
1	1706	1651	ν_{as}(C=C$_{Arom}$)	(3.86 ± 0.13) × 10^{-3}

Supplementary Table 3. Kamlet-Taft and deconvolution parameters for the chemiluminesecnce of LHP in different solvents

	Kamlet-Taft parameters			Deconv. A		Deconv. B	
	α	*β*	*π**	λ_A / nm	I_A	λ_B / nm	I_B
**1-hexanol**	0.8	0.84	0.4	427	0.35510	530	0.96402
**1-octanol**	0.77	0.81	0.4	424	0.25428	531	0.96335
**Acetophenone**	0.04	0.49	0.9	432	0.80763	515	0.50486
**Chlorobenzene**	0	0.07	0.71	432	0.75681	515	0.53506
**DMA**	0	0.76	0.88	432	0.67778	512	0.71819
**DMF**	0	0.69	0.88	427	0.32782	519	0.97162
**Dodecane**	0	0	0.05	424	0.69219	521	0.61786
**Hexadecane**	0	0	0.08	432	0.69585	521	0.66176
**Ethylene glycol**	0.92	0.52	0.9	431	0.68178	513	0.74711
**Toluene**	0	0.11	0.54	430	0.75400	516	0.56600
**NMP**	0	0.77	0.92	432	0.04153	524	0.96800

Supplementary Table 4. Rate constants of the solid state thermochemiluminescence reaction of LHP

T / K	k_{obs} / s^{-1}
**423.15 ± 1**	$8.27 \times 10^{-1} \pm 2.89 \times 10^{-2}$
**413.15 ± 1**	$4.03 \times 10^{-1} \pm 1.97 \times 10^{-2}$
**403.15 ± 1**	$1.58 \times 10^{-1} \pm 9.14 \times 10^{-3}$
**393.15 ± 1**	$6.35 \times 10^{-2} \pm 2.88 \times 10^{-3}$
**383.15 ± 1**	$1.44 \times 10^{-2} \pm 1.16 \times 10^{-3}$

Supplementary Table 5. Activation parameter of the thermochemiluminescence reaction of LHP in solid state, and in chlorobenzene (CB) and *N*-methyl-2-pyrrolidone (NMP) solutions

	Solid	CB	NMP
E_a / kJ mol^{-1}	113.45 ± 4.61	88.41 ± 8.56	50.52 ± 0.30
$\Delta H^‡$ / kJ mol^{-1}	114.61 ± 3.44	85.25 ± 8.58	47.41 ± 0.28
$\Delta S^‡$ / J K^{-1} mol^{-1}	21.61 ± 0.82	–77.83 ± 14.93	–159.36 ± 3.14
$\Delta G^‡$ / kJ mol^{-1} (at 273 K)	108.71 ± 3.66	106.51 ± 12.66	90.94 ± 1.13

Supplementary Table 6. Rate constants and *Q* values extracted from the isothermal kinetic measurements

Temperature / °C	k_{obs} / min^{-1}	Q_{10}
**50**	$2.49 \times 10^{-6} \pm 1.06 \times 10^{-9}$	7.28
**60**	$8.80 \times 10^{-6} \pm 8.28 \times 10^{-9}$	2.25
**70**	$1.41 \times 10^{-5} \pm 1.86 \times 10^{-8}$	4.40
**80**	$6.92 \times 10^{-5} \pm 8.21 \times 10^{-8}$	

Supplementary Table 7. **Atomic coordinates**

LHP

O	3.59386600	5.45152200	3.07696700
O	4.81952300	5.18182700	2.34461800
C	5.95202800	2.47080500	5.72343100
C	2.47837500	1.20244400	1.87984300
H	3.11285300	0.50392300	2.41442900
C	6.35140800	1.13700000	5.53875800
H	5.86023800	0.54055000	4.77857900
C	2.48357000	2.55768500	2.26911500
C	1.67323800	0.77536900	0.82896800
H	1.67401700	-0.27104100	0.53826200
C	1.66832000	3.47260100	1.57653600
H	1.67744900	4.51981300	1.85401000
C	6.58071700	3.25126500	6.71040700
H	6.25824600	4.27751900	6.85098800
C	0.86459800	1.69291400	0.14561200
H	0.23809500	1.35804900	-0.67621800
C	7.99173400	1.37583600	7.30309000
H	8.78185800	0.95188200	7.91634500
C	7.36859900	0.59495800	6.32645500

H	7.67337400	-0.43685000	6.17797100
C	7.59463300	2.70557700	7.49320000
H	8.07513500	3.31279500	8.25479600
C	0.86669800	3.03854100	0.52014100
H	0.24582200	3.75337300	-0.01137700
N	4.21772300	2.22059000	3.93932100
N	4.50603200	4.28602800	4.95125200
C	3.38274400	4.39370800	4.01889200
C	3.34795600	2.98981200	3.36795400
C	2.12869500	4.80437000	4.78822400
C	4.88529200	3.04991900	4.89398800
C	1.05673600	3.93058500	5.00781800
H	1.06757500	2.92888400	4.59295500
C	2.08856800	6.09319900	5.34364300
H	2.92485600	6.76528300	5.18854700
C	-0.04165200	4.34100000	5.76899800
H	-0.86481200	3.65214700	5.93445900
C	0.98515800	6.50391100	6.09047600
H	0.96296200	7.50562900	6.50972500
C	-0.08344300	5.62797600	6.30739500
H	-0.94032600	5.94664400	6.89390500
H	5.48895100	5.48928300	2.98275200

Lophine

N	1.086400	-0.899900	-0.022300
H	2.042700	-1.207900	0.070100
N	-1.121700	-0.939100	0.000500
C	-0.033100	-1.691300	-0.003200
C	-0.704800	0.373700	-0.007200
C	0.689200	0.426800	-0.002900
C	-0.011200	-3.156900	0.006100
C	1.185300	-3.891500	-0.069300
H	2.143700	-3.385400	-0.151400
C	1.166400	-5.286700	-0.050700
H	2.101100	-5.836600	-0.110700
C	-0.047200	-5.972800	0.040900
H	-0.061300	-7.058600	0.054700
C	-1.243000	-5.249700	0.112400
H	-2.191600	-5.774300	0.182000
C	-1.228300	-3.856900	0.095800
H	-2.150300	-3.288500	0.149900
C	-1.695900	1.465000	-0.052700
C	-2.948600	1.285200	0.560700
H	-3.154800	0.345300	1.061600
C	-3.912300	2.293300	0.522500
H	-4.872800	2.136600	1.005600
C	-3.647000	3.499100	-0.133900
H	-4.398000	4.283300	-0.164700
C	-2.410400	3.682800	-0.759400

H	-2.199200	4.608500	-1.287500
C	-1.445200	2.675200	-0.723100
H	-0.498300	2.823300	-1.231900
C	1.683300	1.507100	0.061800
C	1.502900	2.606500	0.921700
H	0.607500	2.663100	1.531800
C	2.466200	3.611200	1.002100
H	2.308900	4.450900	1.673000
C	3.634900	3.535600	0.236900
H	4.385000	4.317900	0.304800
C	3.829400	2.446300	-0.615800
H	4.729000	2.380900	-1.221200
C	2.861900	1.443500	-0.706100
H	3.007800	0.619200	-1.399600

Lophine anion

C	-3.159900	-0.361600	-0.259100
C	1.509200	-2.849600	-0.908200
H	0.578100	-3.156500	-1.373200
C	-3.751100	-1.642100	-0.249300
H	-3.096300	-2.507200	-0.273600
C	1.570100	-1.557900	-0.339500
C	2.603600	-3.712900	-0.868700
H	2.524000	-4.699800	-1.320100
C	2.775900	-1.186800	0.292100
H	2.845500	-0.216700	0.773000
C	-4.014200	0.760100	-0.224600
H	-3.563200	1.747100	-0.234100
C	3.800100	-3.319500	-0.256400
H	4.654500	-3.991300	-0.227300
C	-5.976100	-0.671200	-0.172800
H	-7.056600	-0.789800	-0.139900
C	-5.137200	-1.792500	-0.206900
H	-5.567700	-2.792000	-0.199600
C	-5.399900	0.605600	-0.182100
H	-6.036900	1.487800	-0.156700
C	3.872900	-2.049600	0.326900
H	4.786600	-1.731400	0.824700
N	-0.857500	-1.257200	-0.352400
N	-1.103100	1.010500	-0.299200
C	0.228600	0.725500	-0.356500
C	0.382500	-0.692100	-0.373300
C	1.197900	1.826200	-0.456800
C	-1.703300	-0.202200	-0.303800
C	2.416800	1.716900	-1.159000
H	2.665300	0.779900	-1.646300
C	0.893600	3.079900	0.118600
H	-0.053200	3.183800	0.638400
C	3.298500	2.794900	-1.255300

H	4.228500	2.675900	-1.807500
C	1.772800	4.157500	0.018400
H	1.509200	5.108400	0.477300
C	2.988400	4.024900	-0.664400
H	3.675000	4.864400	-0.741500

Supplementary Figures

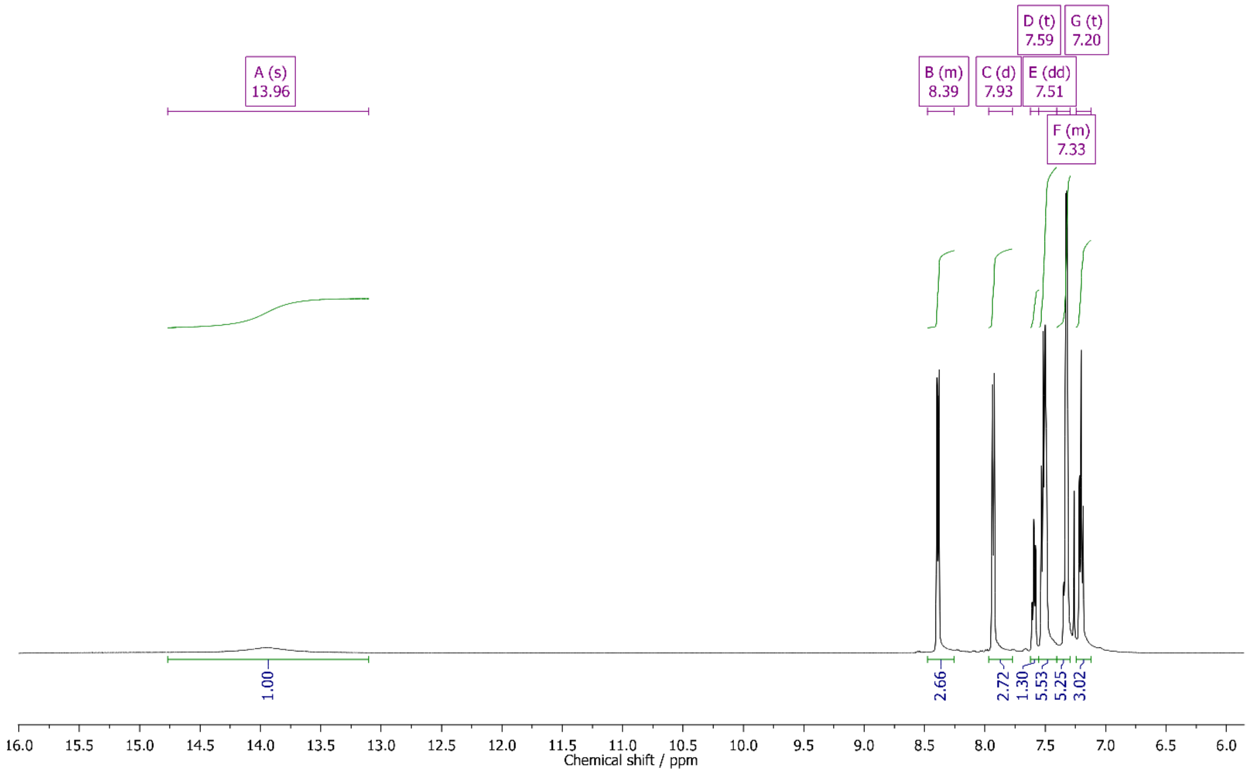

Supplementary Figure 1. ^{1}H NMR spectrum of lophine hydroperoxide.

A

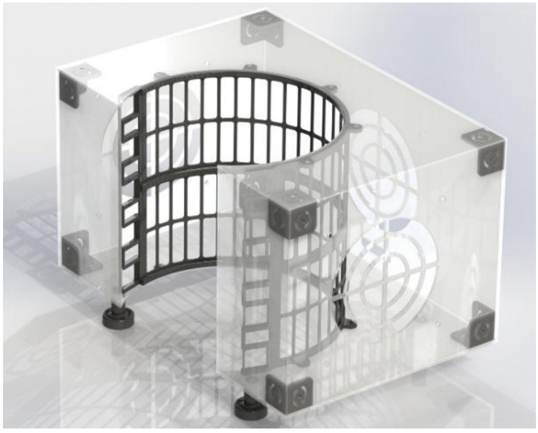

B

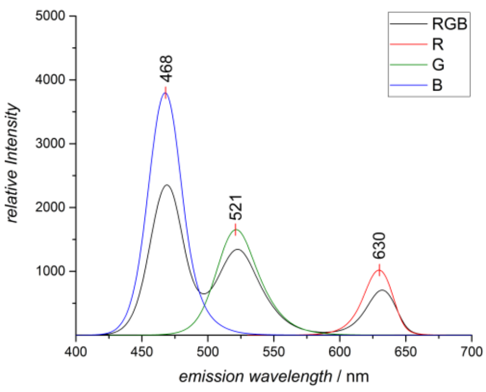

Supplementary Figure 2. 3D model of the cooled sample compartment of the RGB photoreactor (A) and the emission spectra of its LEDs (B). R. G and B stand for red, green and blue contributions to the spectrum.

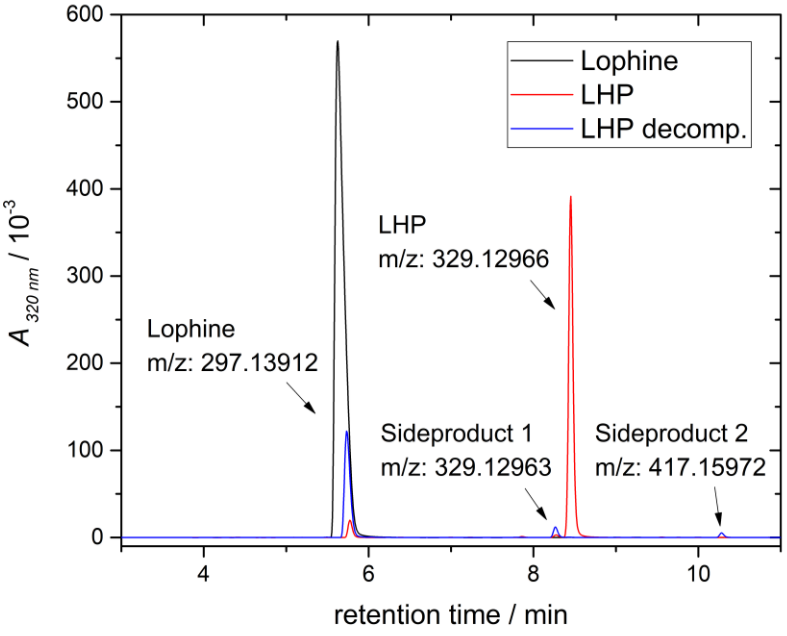

Supplementary Figure 3. Analytical UHPLC chromatograms of lophine, lophine hydroperoxide (LHP) and the decomposition products of LHP ('LHP decomp').

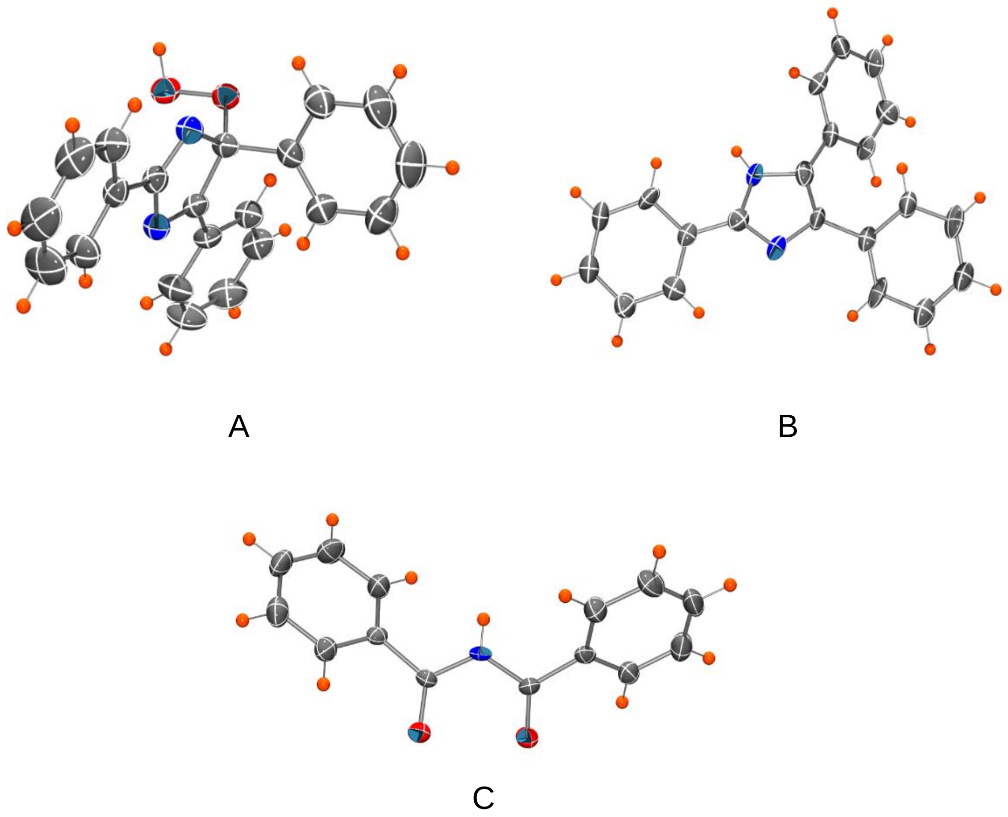

Supplementary Figure 4. ORTEP diagrams, shown at 50% probability level, of the molecular structures of LHP (A), lophine (B) and *N*-benzoylbenzamide (C) in the respective crystals. The carbon, oxygen and nitrogen atoms are represented as gray, red and blue thermal ellipsoids. The hydrogen atoms are represented as red spheres of arbitrary radius.

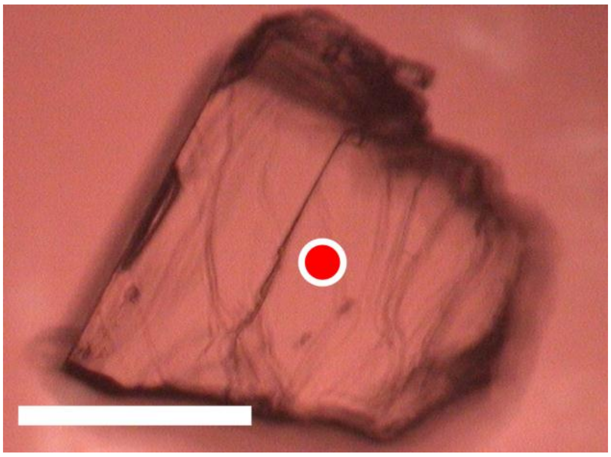

Supplementary Figure 5. Representative image of LHP crystal from which the IR spectra were recorded at 20 ºC, shown at 8-fold magnification. The red spot represents the focus point of the IR radiation. The length of the scale bar is 50 μm.

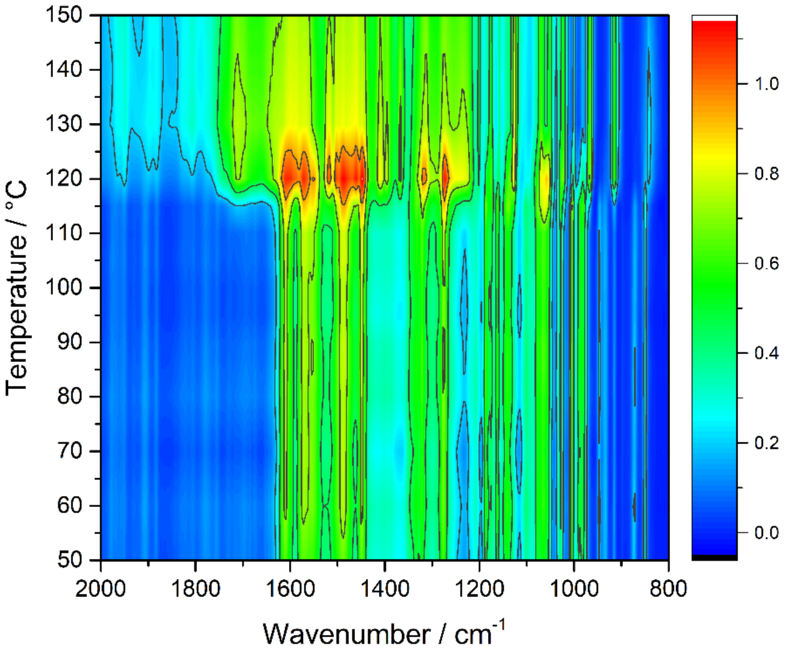

Supplementary Figure 6. IR spectrum of a heated crystalline sample of LHP, showing changes due to decomposition around 115 °C. The color-coded scale given on the right corresponds to intensity in arbitrary units.

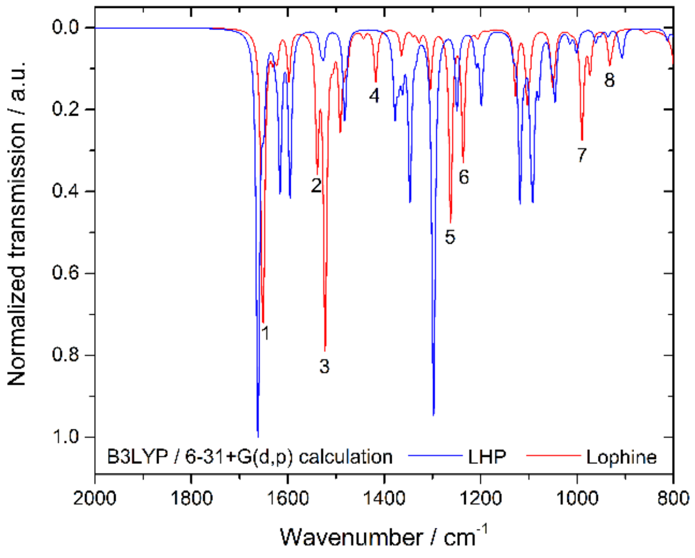

Supplementary Figure 7. Calculated IR spectra of lophine and lophine hydroperoide (LHP) used to assign the characteristic vibrations of lophine that was obtained as a product of the reaction. The ordinal numbers of the selected bands refer to the details in Supplementary Table 2.

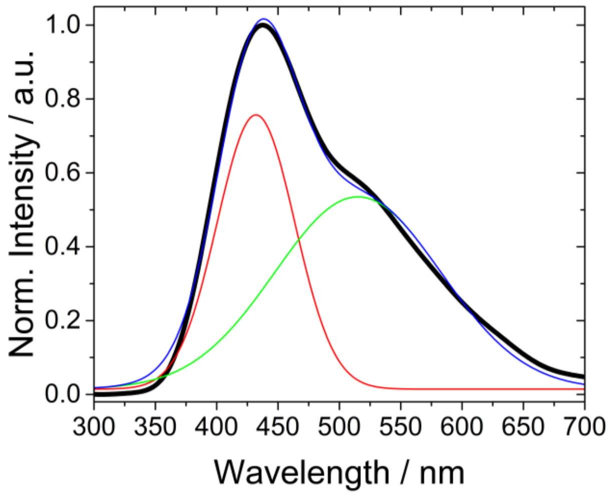

Supplementary Figure 8. Gaussian deconvolution of the chemiluminescent emission spectra of LHP in chlorobenzene. Black line – experimental spectrum, blue line – reconstructed spectrum, red and green lines – component bands.

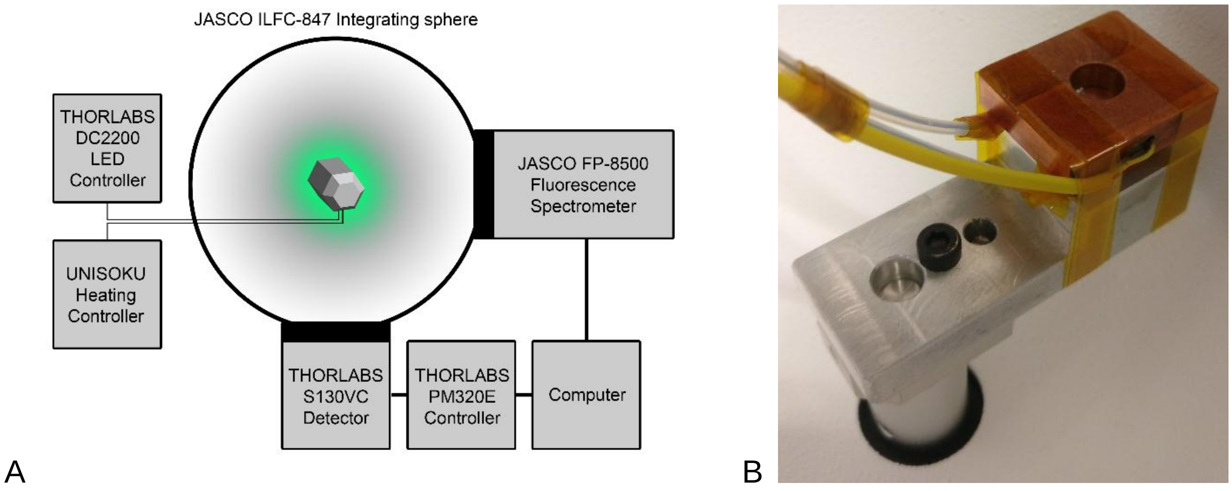

Supplementary Figure 9. Experimental setup used for determination of the chemiluminescence quantum yield. (A) Schematic illustration of the setup with the optical and electronic components. (B) Custom-made micro-heating stage used for the solid-state chemiluminescence experiments.

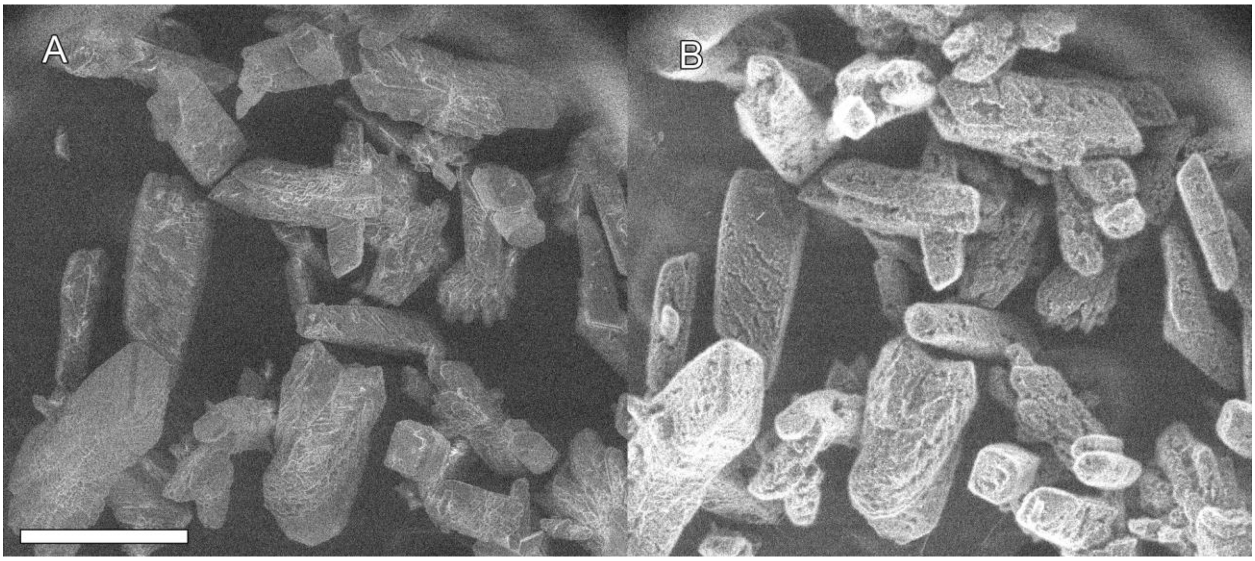

Supplementary Figure 10. SEM micrographs of LHP crystals (A) at room temperature and (B) after decomposition at 160 °C. The length of the scale bar (applies to both panels) is 300 μm.

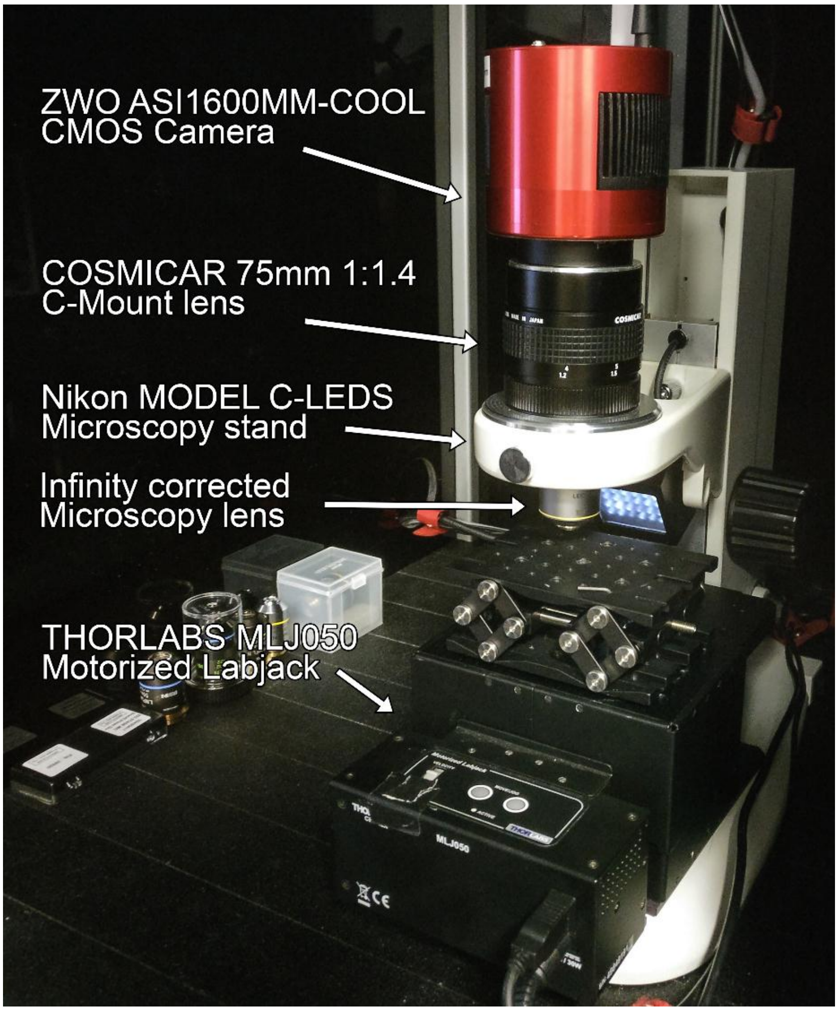

Supplementary Figure 11. Low-light microscopy setup used for visualization of the solid-state thermochemiluminescence.

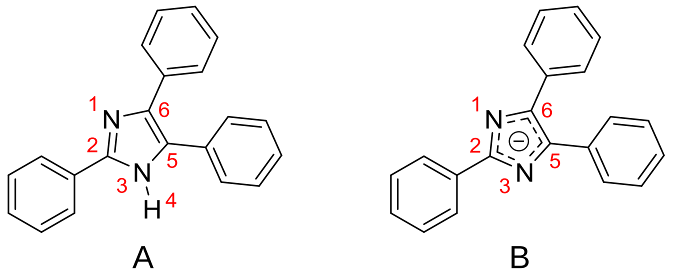

	Lophine	Lophine anion
**N1**	-0.33198	-0.39194
**C2**	0.18092	0.14832
**N3**	-0.21928	-0.39197
**H4**	0.28520	-
**C5**	0.02888	0.01452
**C6**	0.05875	0.01449

Supplementary Figure 12. IBO charge analysis of lophine (A) and the lophine anion (B).

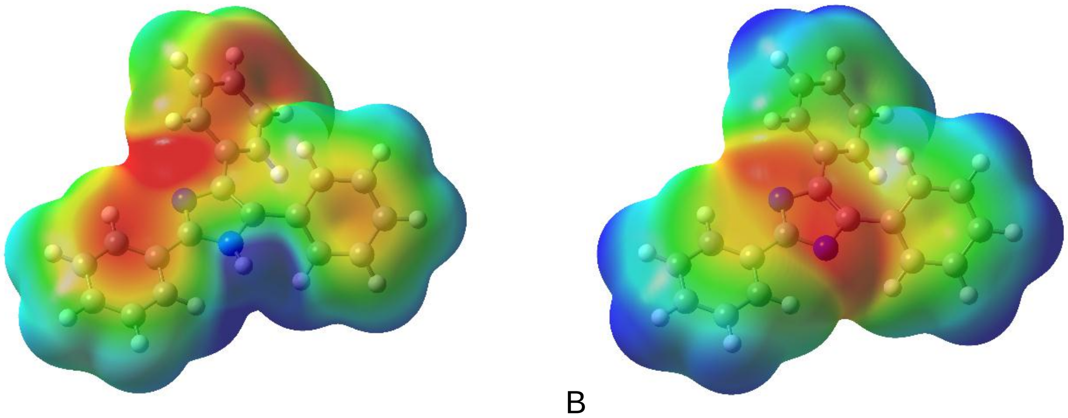

A B

Supplementary Figure 13. ESP mapped on the surface of the total density of lophine (A) and the lophine anion (B). Colors are linearly distributed, red to blue, from -2.0×10^{-2} to 2.5×10^{-2} for lophine (A) and -0.15 to -7.0×10^{-2} for the lophine anion (B). The color-coding scale is identical to that shown in Figure 4B in the main text.

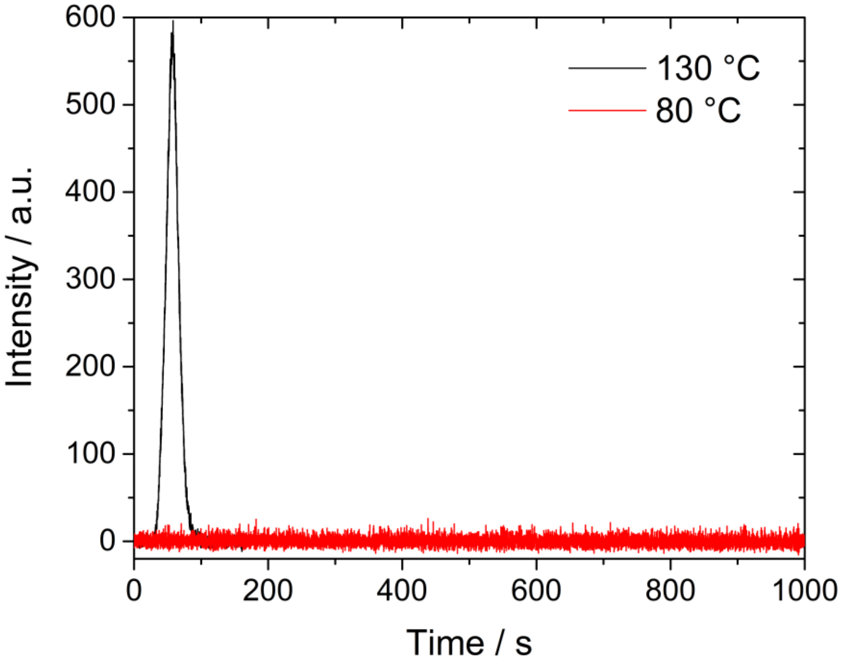

Supplementary Figure 14. Kinetic traces of the thermochemiluminescence reaction of LHP crystals at 130 °C and 80 °C.

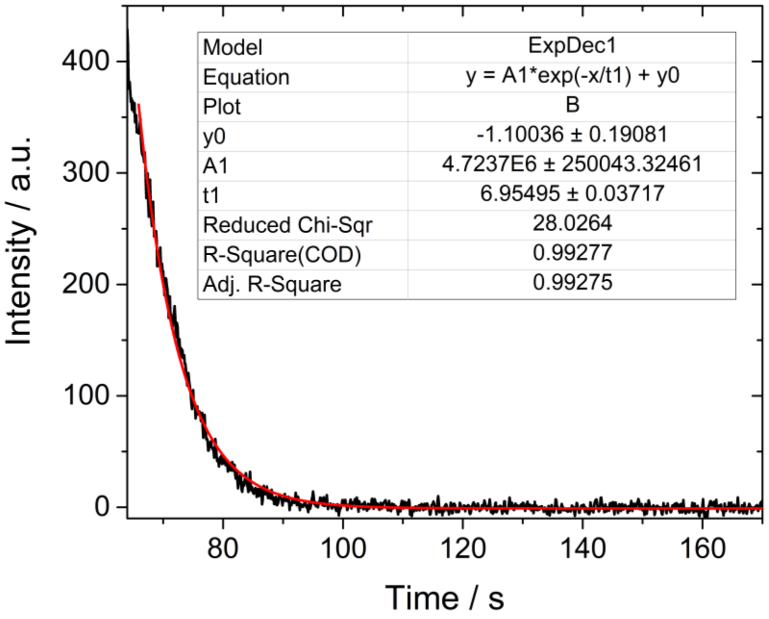

Model	ExpDec1
Equation	y = A1*exp(-x/t1) + y0
Plot	B
y0	-1.10036 ± 0.19081
A1	4.7237E6 ± 250043.32461
t1	6.95495 ± 0.03717
Reduced Chi-Sqr	28.0264
R-Square(COD)	0.99277
Adj. R-Square	0.99275

Supplementary Figure 15. Kinetic traces of the thermochemiluminescence reaction of LHP crystals at 130 °C (black line) and the fit with a first order kinetic model (red line). The fitted parameters are given on the plot.

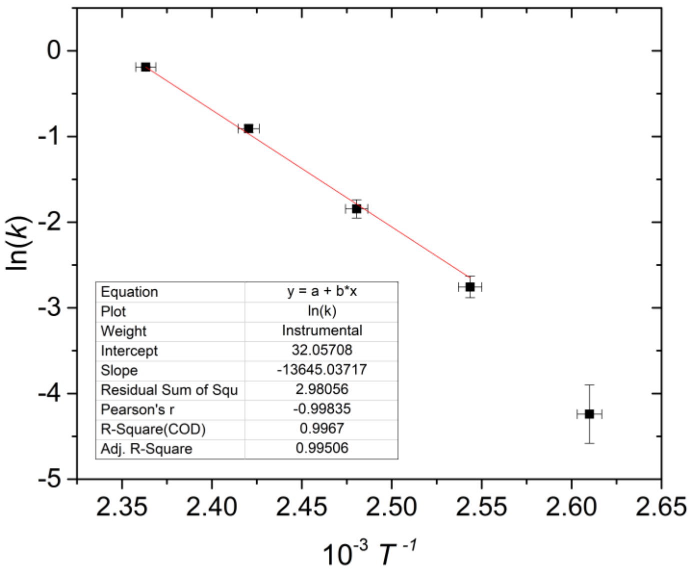

Supplementary Figure 16. Arrhenius plot for the solid-state thermochemiluminescence reaction of LHP. The red line represents the best linear fit. The fitted parameters are given on the plot.

Supplementary References

1 White, E. H. & Harding, M. J. C. The chemiluminescence of lophine and its derivatives. *J. Am. Chem. Soc.* **86**, 5686–5687 (1964).

2 APEX2, Version 2008.3-0 / 2.2-0, Bruker AXS, Inc., Madison, WI, USA (2007).

3 Bruker, SAINT, version 7.60a. Bruker AXS Inc., Madison, WI, USA (2006).

4 Bruker, SADABS, version 2.05. Bruker AXS Inc., Madison, WI, USA (2006).

5 Sheldrick, G. A short history of SHELX. *Acta Crystallogr. A* **64**, 112–122 (2008).

6 Dolomanov, O. V., Bourhis, L. J., Gildea, R. J., Howard, J. A. K. & Puschmann, H. OLEX2: A complete structure solution, refinement and analysis program. *J. Appl. Crystallogr.* **42**, 339–341 (2009).

7 Macrae, C. F. *Et al.* Mercury CSD 2.0—new features for the visualization and investigation of crystal structures. *J. Appl. Crystallogr.* **41**, 466–470 (2008).

8 Barbour, L. J., X-Seed, graphical interface to SHELX-97 and POV-Ray, program for better quality of crystallographic figures; University of Missouri-Columbia, Columbus, MO, USA (1999).

9 POV-Ray for windows, Persistence of vision; Raytracer Pty Ltd, Victoria, Australia (2004).

10 Kamlet, M. J., Abboud, J. L. M., Abraham, M. H. & Taft, R. W. Linear solvation energy relationships. 23. A comprehensive collection of the solvatochromic parameters, π^*, α, and β, and some methods for simplifying the generalized solvatochromic equation. *J. Org. Chem.* **48**, 2877–2887 (1983).

11 Kim, T. J., Tuerkcan, S., Ceballos, A. & Pratx, G. Modular platform for low-light microscopy. *Biomed. Opt. Express* **6**, 4585–4598 (2015).

12 Kim, T. J., Türkcan, S. & Pratx, G. Modular low-light microscope for imaging cellular bioluminescence and radioluminescence. *Nat. Protocols* **12**, 1055–1076 (2017).

13 Frisch, M. J. T., *et al.* Gaussian, Inc, Wallingford, CT, USA (2009).

14 GaussView, version 5, Dennington, R., Keith, T. & Millam, J., Semichem Inc., Shawnee Mission, KS, USA (2009).

15 Becke, A. D. Density-functional thermochemistry. III. The role of exact exchange. *J. Chem. Phys.* **98**, 5648–5652 (1993).

16 Lee, C., Yang, W. & Parr, R. G. Development of the Colle-Salvetti correlation-energy formula into a functional of the electron density. *Phys. Rev. B* **37**, 785–789 (1993).

17 Knizia, G. Intrinsic atomic orbitals: An unbiased bridge between quantum theory and chemical concepts. *J. Chem. Theory t.* **9**, 4834–4843 (2013).

18 Knizia, G. & Klein, J. E. M. N. Electron flow in reaction mechanisms—revealed from first principles. *Angew. Chem. Int. Ed.* **54**, 5518–5522 (2015).
