# Supplementary material for: Thermochemiluminescent peroxide crystals
Source: Nat Commun. 2019 Mar 1;10:997. doi: 10.1038/s41467-019-08816-8 (PMC6397279; doi:10.1038/s41467-019-08816-8)
Supplement: Supplementary file 3 — Description of Additional Supplementary Files [file 41467_2019_8816_MOESM3_ESM.pdf]

Description of Additional Supplementary Files

File Name: Supplementary Movie 1.
Description: Monochromic movie of the solid-state chemiluminescence of a large crystalline aggregate of LHP recorded normal to the solid surface.

File Name: Supplementary Movie 2
Description: Monochromic movie of the solid-state chemiluminescence of several LHP crystals recorded normal to the solid surface.

File Name: Supplementary Movie 3
Description: Monochromic movie of the solid-state chemiluminescence of polycrystalline LHP sample recorded normal to the solid surface.

File Name: Supplementary Movie 4
Description: Color movie of the solid-state chemiluminescence of LHP crystal recorded laterally.

File Name: Supplementary Movie 5
Description: Vertical slicing through a CT scan of partially reacted crystal of LHP.

File Name: Supplementary Movie 6
Description: Disintegration and evolution of gas during heating a crystal of LHP submerged into a drop of oil from room temperature to 150 °C.

File Name: Supplementary Movie 7
Description: SEM micrographs of heated LHP crystals from room temperature to 160 °C.
